# Father Trait Anger and Exposure to Infant Cry: Effects on Emotion, Appraisals of Infants, and Cognitive Performance

**DOI:** 10.1111/jopy.13029

**Published:** 2025-05-23

**Authors:** Lauren M. Francis, Bridgette E. Speranza, Liam G. Graeme, Ashlee Curtis, Peter G. Enticott, Jacqui A. Macdonald

**Affiliations:** ^1^ SEED Lifespan Strategic Research Centre, School of Psychology, Faculty of Health Deakin University Burwood Victoria Australia; ^2^ Centre for Adolescent Health, Murdoch Children's Research Institute Royal Children's Hospital Melbourne Victoria Australia; ^3^ Department of Paediatrics, Faculty of Medicine, Dentistry and Health Sciences University of Melbourne Parkville Victoria Australia

**Keywords:** anger, cognition, fatherhood, impulse control, mentalizing

## Abstract

**Objective:**

Trait anger can impact emotional states, appraisals of others, and cognition. The study aim was to assess in fathers whether these associations are exacerbated by infant crying.

**Method:**

Three hundred sixty‐eight fathers were randomly assigned to infant cry, infant babble, or a non‐infant‐related control while completing assessments of cognitive scope, impulse control, or mentalizing. Trait anger (pre‐exposure), emotional state (pre‐ and post‐exposure), and appraisals of the infant (post‐exposure) were assessed.

**Results:**

Regression analyses revealed that trait anger was associated with increased angry emotional state post‐exposure, including feeling like yelling at someone, feeling like hitting someone, and with negative appraisals of infant temperament. Fathers exposed to cry were more likely to feel angry and like yelling at someone post‐exposure than fathers exposed to babble or pink noise, and appraised the infant more negatively and as having less positive intent than fathers exposed to babble. Neither trait anger nor sound condition were associated with cognitive scope, impulse control, or mentalizing performance. No significant interaction effects between trait anger and infant cry condition were found on any of the dependent variables.

**Conclusions:**

Fathers may benefit from support to modulate their responses to infant cry. Fathers with higher trait anger may benefit from intervention to manage responses to both positive and negative infant expressions.

## 1 Introduction

Trait anger is the dispositional characteristic of individuals to experience, with or without provocation, feelings of anger from mild frustration to intense rage (Spielberger et al. 1995). Trait anger increases the frequency and intensity of angry emotional states (i.e., anger felt in the moment), elevates the likelihood of hostile social appraisals, and narrows cognition (Gable et al. 2015; Li and Xia 2020; Spielberger et al. 1995). For parents of infants, trait anger may therefore present a risk to sensitive caregiving (i.e., the father's ability to accurately interpret his infant's behavior and respond promptly and appropriately; Ainsworth et al. 1974), particularly in the context of early parental stressors.

Crying is the first communication strategy that infants use to express their needs to caregivers (LaGasse et al. 2005). Indeed, recent research with fathers exposed to infant distress suggests crying is interpreted as meaningful communication, evident in neural activation in brain regions linked to social–emotional processing (Khoddam et al. 2020). However, infant cry is also a commonplace parental stressor. For parents with high levels of trait anger, exposure to a crying infant may evoke trait characteristics that interfere with optimal parenting. In a study of mothers who were exposed to 10 min of a recorded infant cry, trait anger predicted unwanted and intrusive thoughts of harm toward the crying infant (Fairbrother et al. 2015). To the authors' knowledge, this is the only study that has investigated trait anger as a predictor of parent responses to infant distress. In adjacent literature, higher levels of mother trait negative emotions have been associated with more negative emotions after exposure to infant cry and more negative attributions about the crying infant (Leerkes et al. 2015). Notably, in fathers, after exposure to infant cry, state anger (trait anger was not assessed) was associated with thoughts of infant‐related harm (Fairbrother et al. 2019). Further, in fathers, trait anger has been prospectively linked to poorer bonding with infants and to increased parenting stress when children reach their toddler years (Francis et al. 2023).

In general, intense and poorly controlled anger is more prevalent in men compared to women (Okuda et al. 2015). Gender differences are also evident in physiological, neurological, and emotional responses to infant cries, supporting the importance of gender‐specific investigations (Out et al. 2010; Rigo et al. 2017; Roellke et al. 2019; Sander et al. 2007). Fathers are increasingly involved in day‐to‐day caregiving of children, and fathers' sensitive provision of care is important for child behavioral, cognitive, and emotional and development (Bakermans‐Kranenburg et al. 2019; Cooke et al. 2022; Rodrigues et al. 2021). Further, little is known about the cognitive mechanisms by which trait anger may impact fathering particularly when exposed to stressors. The provision of sensitive caregiving relies on parental emotion regulation, accurate appraisal of the infant, and access to cognitive resources to attend to infant needs (Rutherford et al. 2015; Zeegers et al. 2017). Knowledge on the potential of trait anger to undermine these is critical to inform future father‐focused interventions and parenting supports. Here we investigate the roles of father trait anger and exposure to infant cry on three critical indicators of sensitive parenting: emotional state, appraisals of an infant's temperament and intent, and cognitive performance.

### 1.1 Impacts of Trait Anger and Infant Crying in The Family Context

#### 1.1.1 Angry Emotional State

Trait anger involves tendencies to interpret stimuli as hostile, to attend to and ruminate on the perceived hostile stimuli, and to fail to recruit and engage in effortful control to regulate and negate those tendencies (for a review see; Wilkowski and Robinson 2010). Through these mechanisms, trait anger impacts the frequency and intensity of angry emotional states (Spielberger 1999; Wilkowski and Robinson 2010). Anger serves as motivation for an individual to persevere and resolve impediments to goal pursuit (Carver and Harmon‐Jones 2009; Schmitt et al. 2019). For example, feeling anger in response to a physical threat can motivate aggression for self‐preservation (Wilkowski and Robinson 2010); however, in the parenting environment, where the stressor that elicits or maintains an angry emotional state is an inconsolable crying infant, goal pursuit motivated by anger may lead to problematic or insensitive responses. Feelings of anger toward a crying infant are reported by parents across the existing literature (Muller et al. 2023), though notably men tend to find infant cries more aversive and show greater heart rate increases in response to infant cries than women (Out et al. 2010; Zeifman 2003). Further, the duration of exposure to the sound of a crying infant is positively associated with increases in physiological arousal (Out et al. 2010). In the case of a father with high levels of trait anger, an infant that is not able to be immediately consoled and that continues to cry may prompt further ruminative attention, prolonging an angry emotional state. This may create a feedback loop where, due to feelings of anger, the father is less able to provide sensitive care, and therefore may be less likely to soothe the infant, resulting in continued and potentially amplified feelings of anger.

#### 1.1.2 Appraisals of Infants

Anger increases the likelihood of automatically appraising others as harboring hostile intentions (Li and Xia 2020). It is plausible that this also applies to appraisals of infants. For example, in a study of childless young adults, experiencing anger was associated with increased appraisals of a crying child as being more negative and intentionally difficult (Cohen‐Bendahan et al. 2014). The cry of an infant is argued to have been evolutionarily refined to provoke adverse affective responses in caregivers to motivate them to alleviate their discomfort through the provision of care to the infant (LaGasse et al. 2005). However, in fathers with elevated levels of trait anger, the adverse response to an infant's cry may be amplified, eliciting default trait hallmarks of negative and hostile appraisals. Paternal appraisals of infants are particularly important to investigate, as negative appraisals of infant temperament are markers of impaired father‐infant bonds and predict subsequent parenting outcomes such as harsher parenting behaviors (Berlin et al. 2013; Condon et al. 2008).

#### 1.1.3 Cognitive Performance

In prior studies, trait anger has been associated with poorer cognitive performance (Fonagy et al. 2016; Gable et al. 2015; Lievaart et al. 2018). This may be particularly pronounced in the context of infant cry, which may be interpreted by those high in trait anger as signaling threat or hostility. One explanation for poorer cognition is linked to the purpose of anger, that is, to motivate a resolution to the threat or obstacles to goal pursuit (for a review see: Carver and Harmon‐Jones 2009). When anger is elicited, cognitive resources and attentional scope are narrowed to ignore irrelevant information and facilitate direct approach toward the stimuli that has evoked the anger (Gable et al. 2015). High trait anger is also associated with poor impulse control (i.e., the ability to refrain from a response to complete a goal), especially when primed with the inducement of an angry emotional state (Lievaart et al. 2018). Poor impulse control may present a particularly heightened risk to family safety among parents with high trait anger who, in prior research, have been found to be more likely to report unwanted thoughts of harm when hearing an infant cry (Fairbrother et al. 2015, 2019). Further, a connectome‐wide association analysis of trait anger found that trait anger modulates functional connectivity to brain regions associated with increased approach motivation and poorer inhibitory control (Kim et al. 2022). Trait anger is also associated with poorer self‐reported mentalizing (i.e., the ability to interpret and understand the mental states and emotions of oneself and others; Fonagy et al. 2016; Francis et al. 2024). Less is known about links between trait anger and performance‐based mentalizing tasks. Further understanding here is important because parental mentalizing ability is a contributing factor in sensitive caregiving behavior (Camoirano 2017) and, when assessed from coded interviews, is shown in meta‐analyses to be linked to intergenerational transmission of attachment security (Zeegers et al. 2017). Examination of how father trait anger and infant cry affect performance‐based assessments of cognition remains unexplored and could extend knowledge on the nature of these associations.

### 1.2 The Current Study

The primary aim of the current study was to investigate whether exposure to infant cry exacerbates effects of trait anger on fathers' angry emotional state (feel like expressing anger verbally, feel like expressing anger physically, and feeling angry), appraisals of infants (negative temperament, negative intent, and positive intent), and cognitive performance (cognitive scope, impulse control, and mentalizing). We first hypothesized that trait anger and exposure to infant cry, relative to control conditions, would each be positively associated with post‐exposure feelings of anger, feeling like yelling at someone, and feeling like hitting someone, and that effects of trait anger would be stronger for fathers in the infant cry condition. Second, we hypothesized that trait anger and exposure to infant cry, relative to control conditions, would be positively associated with post‐exposure negative appraisals of the temperament of the infant and appraisals of negative intent of the infant, and negatively associated with appraisals of positive intent of the infant, and that effects of trait anger would be stronger for fathers in the infant cry condition. Third, we hypothesized that trait anger and exposure to infant cry, relative to control conditions, would be associated with cognitive performance (i.e., narrowed cognitive scope, poorer impulse control, and poorer mentalizing ability), and that effects of trait anger would be stronger for fathers in the infant cry condition. Study hypotheses and methods were preregistered via the Open Science Framework (https://osf.io/x24az/registrations). Minor adaptations were made (for explanations of the adaptations, see File S1).

## 2 Method

### 2.1 Participants

Participants were recruited in August and September of 2023 through the online recruitment program Prolific, where they were pre‐screened for the following eligibility criteria: (1) male, (2) right‐handed, (3) aged 18–60, (4) father of between one and five children, (5) currently living with at least one biological child, (6) youngest child 10 years old or younger, (7) fluent in English, (8) normal or corrected to normal vision, and (9) no hearing loss or hearing difficulties. Participants were required to complete the study on a computer with headphones and to download Inquisit.

An a priori power analysis conducted in G*Power version 3 found that an overall sample size of 333 would be adequate to address the research questions specified in this study. Specifically, a sample size of 37 participants per task condition and sound condition combination would provide 80% power to detect a small interaction effect (*F* ^2^ = 0.11, alpha = 0.05), with up to five predictor variables (covariates) included in the model.

Participants were included in the analytic sample if they met eligibility criteria, completed all trait anger, cognitive, and emotional state assessments, and passed all data quality checks (see File S2 for details of data quality checks). For a flow chart summary of participant exclusions, see File S3. Briefly, of 605 individuals who provided consent, 416 completed pre‐exposure measures and correctly completed the headphone set‐up task and thus were randomized to condition, and 48 were removed during quality checks. The resulting analytic sample included 368 participants, with 31–54 participants in each sound condition by cognitive task group (see File S2 for the number in each group). We note that this number is slightly lower per group than required for power (outlined above) due to the use of randomized allocation without replacement.

Participants were on average 37.30 years of age (SD = 6.71). Participants had an average of 1.76 children (SD = 0.83) and had youngest children between 2 weeks and 10 years of age (*M* = 4.59, SD = 2.88). For 12% of the sample (*n* = 44) that youngest child was an infant 1 year old or younger. Trait anger scores (*M* = 16.47, SD = 4.65, *N* = 368) were not significantly different from the norm for males 30 years and older (*M* = 16.80, SD = 4.24, *n* = 124; Spielberger 1999), *t*(490) = −0.698, *p* = 0.486. For more information about participant characteristics, see Table 1.

**TABLE 1:** Participant characteristics.

Variable	*n*	%
Biological parent of youngest child	365	99
Primiparous	160	43
In a relationship	360	98
Post‐high school qualification	313	85
Residence^a^		
United Kingdom	149	40
Other Europe	81	22
North America	87	24
Africa	36	10
Australia and New Zealand	7	2
Asia	4	1
South America	2	1
Middle East	2	1
Income above median^b^	231	63
Subjective social status above average	226	61
Participation environment		
Alone at home/work	210	57
With quiet others at home/work/in public	156	42
With noisy others at home	2	1

*Note:* ^a^= Due to rounding percentages may not sum to 100; ^b^= Income above the median for own country of residence.

### 2.2 Procedure

**FIGURE 1:**
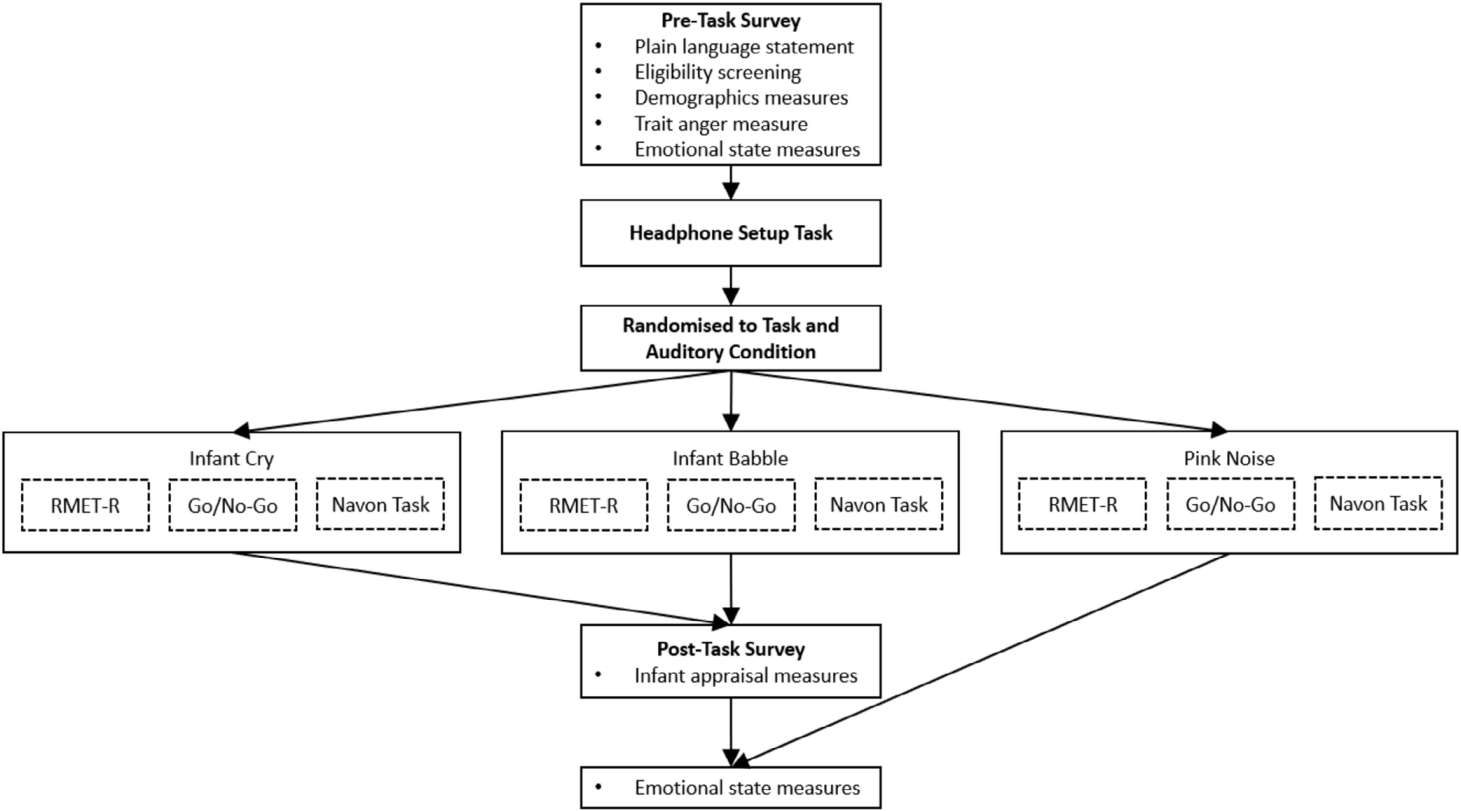
Flow diagram summarizing participant involvement in the study. Participants assigned to the pink noise condition were not exposed to the sound of an infant and thus were not asked to appraise any infant. RMET‐R, Reading the Mind in the Eyes Task—Revised.

For a summary of participant involvement, see Figure 1. This study was a randomized controlled experiment in which participants completed a pre‐exposure survey hosted on Qualtrics (2023) assessing demographics, trait anger, and pre‐exposure angry emotional state. Then, they were redirected to Inquisit (version 6.6.1; Millisecond Software 2022c ) where they completed a procedure to ensure that they were using headphones (sourced from; Millisecond Software 2022d), then they were randomly assigned to one of three cognitive tasks (which they were informed would take approximately 10 min) assessing either cognitive scope (Navon task; Navon 1977), response inhibition (go/no‐go task; Fillmore et al. 2006), or mentalizing (Reading the Mind in the Eyes Task—Revised [RMET‐R]; Baron‐Cohen et al. 2001). Within tasks, participants were further randomized to one of three sound stimuli conditions: infant cry, infant babble, or non‐infant‐related control (pink noise) to which they were exposed throughout the entirety of the assigned cognitive task. Participants were asked to complete only one task while being exposed to only one sound condition to minimize burden. Finally, participants completed the post‐task survey assessing post‐exposure angry emotional state and appraisals of the infant. Participants were compensated with GBP3.00 for approximately 30 min of participation.

### 2.3 Materials

#### 2.3.1 Sound Stimuli

This study included three sound conditions. The exposure condition was an infant cry recording (which included distressed cries from a single infant) and was intended to evoke anger. The infant‐related control condition was operationalized as an infant babble recording (which included happy noises such as coos and babbles from a single infant) and was not intended to induce any negative emotional state. The final condition was a non‐infant‐related control condition operationalized as pink noise, which is a continuous signal sampled from all frequencies audible to the human ear (20–20,000 Hz) with equal energy per octave. Recordings used for the infant cry and infant babble stimuli were sourced from Barr et al. (2014). See Barr et al. (2014) for a characterization of the different cry and babble sounds included in each recording. Pink noise was generated from Audacity version 3.3.2. Participants were informed that the recording would play throughout their completion of the cognitive task, which would take approximately 10 min (whereas in Barr et al. (2014) participants were not advised of the duration). All audio files (available at: https://doi.org/10.17605/osf.io/x24az) were 5‐min long and set to loop.

#### 2.3.2 Measures

##### 2.3.2.1 Participant Characteristics

A range of participant characteristics were collected pre‐exposure. These were father age (years), age of youngest child (years), number of children, in a relationship (0 = *No*, 1 = *Yes*), completion of post‐high school qualification (0 = *No*, 1 = *Yes*), household income (0 = *Below or at national median*, 1 = *Above national median*), subjective social status (0 = *Below perceived average social status*, 1 = *Above perceived average social status*; Adler et al. 2000), country of residence (0 = *Outside of United Kingdom*, 1 = *United Kingdom*), and participation environment (0 = *Participated with others present*, 1 = *Participated alone*).

##### 2.3.2.2 Trait Anger

Trait anger was assessed pre‐exposure with the trait anger scale of the State–Trait Anger Expression Inventory‐2 (STAXI‐2; Spielberger 1999). The trait anger scale is comprised of 10 items which assess tendency to feel anger generally and to respond to provocation with anger. It includes items such as “I feel infuriated when I do a good job and get a poor evaluation.” Response options range from 1 = *Almost never* to 4 = *Almost always*. Higher scores indicate higher levels of trait anger. Validity and reliability of the trait anger scale has been demonstrated in adults and men specifically (García‐León et al. 2002; Lievaart, Franken, et al. 2016; Spielberger 1999). In this sample the trait anger scale had good scale reliability (McDonald's omega = 0.86).

##### 2.3.2.3 Angry Emotional State

To measure emotional state pre‐ and post‐exposure, participants were asked to respond to “how you currently feel on the following” for items “like yelling at somebody,” “like hitting someone,” and “angry,” which were sourced from each of the STAXI‐2 state anger subscales “feel like expressing anger verbally,” “feel like expressing anger physically,” and “feeling angry” subscales, respectively (Spielberger 1999). An additional seven items of other emotion states (e.g., happy) were included to minimize the salience of the angry emotional state variables of interest. Responses were provided on visual analogue scales for each angry emotional state indicator ranging from 0 = *Not at all* to 100 = *A lot*. The three angry emotional state items were all positively skewed at the *p* < 0.001 level (skewness_range_ = 1.21–1.82). In regression analyses, to manage positive skew, anger items were binarized based on original STAXI‐II scale cut offs (where greater than the 75th percentile = higher anger; Spielberger 1999).

##### 2.3.2.4 Appraisals of the Infant: Negative Temperament, Negative Intent, and Positive Intent

Participants in the infant cry and infant babble conditions were asked post‐exposure to appraise the infant in the audio that they heard. Participants were asked to rate how likely it was that the infant had a “hostile,” “negative,” and “difficult” temperament on 10‐point Likert scales from 1 = *Not at all* to 10 = *Extremely likely*. As per Crouch et al. (2008), an average score of the items was used as an appraisal of the infant as having negative temperament. These three items had good scale reliability (McDonald's omega = 0.82). Six additional items (e.g., friendly) were included to minimize the salience of the variables of interest. Participants were then asked to rate how likely it was that the child was “deliberately being difficult or trying to upset others,” and “deliberately being pleasant or trying to bring joy to others” (items adapted from the Paternal Postnatal Attachment Scale; Condon et al. 2008), as indicators of appraised negative and positive intent of the infant, respectively. Response options were on a 10‐point Likert scale from 1 = *Not at all* to 10 = *Extremely likely*.

#### 2.3.3 Cognitive Tasks

Participants were assigned to one of three cognitive tasks: The Navon Task (assessing cognitive scope), a go/no‐go task (assessing impulse control), or the RMET‐R (assessing mentalizing).

##### 2.3.3.1 Cognitive Scope

The Navon Task involves assessment of global and local features (Navon 1977) and has been used as an indicator of cognitive scope (Harmon‐Jones et al. 2013). In this task, participants are asked to position their index fingers on the S and H keys. Participants were then presented with (global) letter shapes (H or S) that are comprised of smaller (local) letter shapes (H or S) for 5000 ms. Some of the global letter shapes were comprised of the same local letter shape (e.g., H made up of smaller Hs) and some were comprised of a different local shape (e.g., S made up of Hs). This task involves two conditions; in the global condition, participants were asked to respond as quickly as possible to the global letter shape (e.g., press the S key if the global shape is an S and the H key if the global shape is an H). In the local condition, participants were asked to respond as quickly as possible to the local letter shape (e.g., press the S key if the local shape was an S and the H key if the local shape was an H). The order that each participant completes the local and global conditions is randomly assigned. A global‐minus‐local condition average latency difference score for correct responses was used as the outcome, in which a more positive score indicated more narrowed/local scope and a more negative score indicated more broad/global scope. The code for this task was adapted from Millisecond Software (2022b).

##### 2.3.3.2 Impulse Control

The go/no‐go task was used as a measure of response inhibition based on Fillmore et al. (2006). In the go/no‐go task, participants are instructed to press the space bar when presented with “go” target of a green rectangle and to withhold from pressing the space bar upon presentation of a “no‐go” target of a blue rectangle. Before the color is revealed, the rectangle, which is either displayed vertically or horizontally, is presented (for 100, 200, 300, 400, or 500 ms) as an outline (referred to as the “cue” in a given trial); then, color is presented for the remaining duration of the trial. The trial length was 1 s or until a response was recorded, whichever came first. Vertical rectangle cues have a high probability (80%) of being a go trial (i.e., green rectangle) and horizontal rectangle cues have a high probability (80%) of being a no‐go trial (i.e., blue rectangle). A higher proportion of commission errors (i.e., errors where the cue is “go” but the target is “no‐go”) indicate poorer response inhibition (Scholten et al. 2019). The code for this task was adapted from Millisecond Software (2022a).

##### 2.3.3.3 Mentalizing

The RMET‐R was used as a social cognitive indicator of the ability to read emotion from limited facial expression and is theorized to capture adult mentalizing, specifically the ability to understand the mental states of *others* (Baron‐Cohen et al. 2001). Participants are presented with an image of the eye region of a face and given four options surrounding the image, one of which is deemed to be the correct emotion depicted in the image. Participants are asked to select which word best describes what the person in the photo is thinking or feeling. For example, an image portraying the emotion of desire included the response options: 1 = *Joking*, 2 = *Flustered*, 3 = *Desire*, and 4 = *Convinced*. Development of RMET‐R involved piloting of items to determine that the correct emotion was able to be determined by a clear majority of healthy control participants (see Baron‐Cohen et al. (2001) for detail of RMET‐R validation). The 36 items were conducted in 17 and 19 item blocks, each with a different image of the eye region of the face depicting a different mental state. A fixation cross accompanied by a one‐second intertrial interval was added between the items. This task is not time‐limited, and participants were able to look up the definitions of the words anytime with an on‐screen link to a dictionary. Higher number of correctly identified expressions indicates stronger mentalizing ability. The code for this task was adapted from Millisecond Software (2022e).

### 2.4 Analytic Strategy

All data cleaning and analyses were conducted in Stata version 18.0. Study data and analysis code are available at https://doi.org/10.17605/osf.io/x24az. A series of regression analyses were conducted to investigate the main and interaction effects of trait anger and sound condition on key outcomes. Outcome variables were post‐exposure angry emotional state (feeling angry, feeling like yelling at someone, and feeling like hitting someone); appraisals of infant (negative temperament, negative intent, and positive intent); and cognitive performance (narrowed cognitive scope in the Navon task, poorer response inhibition in the go/no‐go task, and poorer metalizing ability in the RMET‐R). Specifically, robust regressions (logistic regressions for angry emotional state outcomes and linear regressions for appraisals of the infant and cognitive performance outcomes) were estimated in three sets in which each outcome variable was regressed first onto sound condition, second onto trait anger and sound condition (entered together), and then third onto trait anger, sound condition, and the multiplicative interaction between those two predictors.

In all analyses, sound condition was included as a three‐level factor variable (i.e., 0 = *Infant cry* (reference group), 1 = *Infant babble*, and 2 = *Pink noise*) designated with the Stata prefix “i.” (equivalent to dummy coding). To aid interpretation, all continuous variables were *z*‐score standardized for analysis and effect sizes are presented in results as standardized odds ratios and betas. Models adjusted for characteristics that differed by sound condition. Models predicting each of the post‐exposure angry emotional state outcomes also adjusted for pre‐exposure scores of that emotional state, indicating change in the emotional state (e.g., the model predicting post‐exposure feeling angry adjusted for pre‐exposure feeling angry). Models that included all participants (i.e., analyses where cognitive performance was not an outcome) adjusted for cognitive test condition assignment. For logistic regressions, odds ratios and confidence intervals were inverted (OR_inverted_ = 1/OR_original_) to show risk increase from pink noise/babble to cry. For linear regressions, β‐values and confidence intervals were inverted (*β*_inverted_ = −1 × *β*_original_) and therefore show risk increase from babble to cry. While we acknowledge the number of comparisons conducted here, the alpha threshold of 0.05 was used for significance testing for all analyses, as recent guidance states that when comparisons are interpreted individually, adjustment of the alpha level for each test is not appropriate (Rubin 2021). All analyses were conducted as preregistered, with the exception of analyses predicting post‐exposure state angry emotional state for which minor adaptations were made post preregistration (see File S1 for a summary of hypothesis and analysis adaptations).

## 3 Results

### 3.1 Participant Characteristics by Group

There were no significant differences between sound conditions for trait anger, father age, age of youngest child, relationship status, post‐high school qualification, household income above the median, or UK country of residence, for the total sample, or within any of the three cognitive testing conditions (i.e., Navon task, go/no‐go, and RMET‐R). The number of children differed across sound conditions for the total sample (*p* = 0.004) such that those in the infant cry condition had significantly more children than those in both the infant babble condition (*p* = 0.014) and the non‐infant‐related control (pink noise) condition (*p* < 0.001), and thus was controlled for in all analyses. See Table S1 for key participant characteristics including comparisons between sound conditions. Participants completed the cognitive testing component of the study in an average of 14.93 min (SD = 2.67) in the Navon condition, 14.58 min (SD = 1.32) in the go/no‐go condition, and 5.43 min (SD = 1.92) in the RMET‐R condition. Summary statistics of key outcome variables for participants in each of the experimental conditions can be found in Table 2.

**TABLE 2:** Summary statistics of key outcome variables for participants in each sound condition.

Variables	Infant cry			Infant babble			Pink noise			Total		
	*n*_a_	*n*_b_	%	*n*_a_	*n*_b_	%	*n*_a_	*n*_b_	%	*n*_a_	*n*_b_	%
Angry emotional state												
Feeling angry	104	45	43	140	25	18	124	19	15	368	89	24
Feeling like yelling at someone	104	38	37	140	22	16	124	25	20	368	85	23
Feeling like hitting someone	104	23	22	140	21	15	124	16	13	368	60	17

	*n*_a_	*M*	SD	*n*_a_	*M*	SD	*n*_a_	*M*	SD	*n*_a_	*M*	SD
Infant appraisals												
Negative temperament	104	5.28	2.16	140	2.00	1.17	N/A	N/A	N/A	244	3.40	2.33
Negative intent	104	2.37	2.33	140	1.86	1.59	N/A	N/A	N/A	244	2.07	1.95
Positive intent	104	1.75	1.45	140	6.62	2.97	N/A	N/A	N/A	244	4.55	3.43
Cognitive performance												
Navon task latency difference (ms)	31	−284.96	271.64	49	−154.63	997.82	34	−430.24	643.01	114	−272.27	759.48
Go/no‐go commission errors (%)	37	0.89	1.33	45	1.42	1.41	36	0.98	1.28	118	1.11	1.36
RMET‐R total correct responses	36	24.86	4.51	46	24.17	5.17	54	26.17	5.03	136	25.14	4.99

*Note: n*_a_ = number of participants who responded to that item or completed that task in each sound condition; *n*_b_ = number of participants who scored above 75th percentile for angry emotional state indicator; RMET‐*R* = Reading the Mind in the Eyes Task—Revised, possible scores range from 0 to 36; Go/no‐go Commission Errors = error rate as a percentage for trials in which cue is “go” and target is “no‐go”; Navon task latency difference = global‐minus‐local condition average latency difference score in milliseconds.

### 3.2 Hypothesis Testing

#### 3.2.1 Angry Emotional State

In logistic regressions predicting post‐exposure angry emotional states (see Table 3), we found significant effects of trait anger on feeling angry, feeling like yelling at someone, and feeling like hitting someone, after controlling for the number of children and pre‐exposure levels of the respective outcome variable. Main effects were detected for sound condition on post‐exposure feeling angry and feeling like yelling at someone such that there were higher odds of anger in the cry condition compared to the babble and pink noise conditions. There was no significant difference between sound conditions on feeling like hitting someone. No interaction effects were detected; therefore, tables present only main effects. For the interested reader, these models were repeated as linear regressions using the continuous version of each outcome variable and are presented in Table S2. We note that the direction of effects was consistent across linear and logistic models.

**TABLE 3:** Logistic regressions assessing trait anger and sound condition as predictors of post‐exposure angry emotional state (*N* = 368).

Variables	Model 1				Model 2			
	OR	95% CIs		*p*	OR	95% CIs		*p*
		LL	UL			LL	UL	
Feeling angry								
Trait anger					**1.78**	**1.34**	**2.37**	**< 0.001**
Cry (vs. babble)	**4.52**	**2.44**	**8.35**	**< 0.001**	**4.76**	**2.51**	**9.04**	**< 0.001**
Cry (vs. pink noise)	**4.24**	**2.24**	**8.04**	**< 0.001**	**4.58**	**2.35**	**8.95**	**< 0.001**
Feeling like yelling at someone								
Trait anger					**1.61**	**1.21**	**2.14**	**0.001**
Cry (vs. babble)	**4.16**	**2.12**	**8.15**	**< 0.001**	**4.14**	**2.08**	**8.22**	**< 0.001**
Cry (vs. pink noise)	**2.40**	**1.25**	**4.59**	**0.008**	**2.40**	**1.23**	**4.71**	**0.011**
Feeling like hitting someone								
Trait anger					**1.61**	**1.17**	**2.21**	**< 0.001**
Cry (vs. babble)	1.94	0.90	4.21	0.092	1.89	0.85	4.20	0.119
Cry (vs. pink noise)	1.81	0.84	3.93	0.131	1.75	0.79	3.88	0.165

*Note:* Model 1 included sound condition as a predictor. Model 2 included trait anger and sound condition as predictors. Both models adjusted for characteristics that differed by sound condition (i.e., number of children), the cognitive test the participant completed, and for pre‐exposure scores of the respective emotional state. Outcome variables were binarized for analysis such that 0 = lower anger, 1 = greater than 75th percentile anger. Bolded rows indicate significance < 0.05.

Abbreviations: CI, confidence interval; LL, lower limit; UL, upper limit.

#### 3.2.2 Appraisals of the Infant

In linear regressions (see Table 4), we found a significant main effect of trait anger on appraisal of infant negative temperament, but no evidence of an effect on appraisals of infant negative or positive intent. We found main effects of sound condition on appraisals of infant negative temperament and appraisals of infant positive intent such that those in the cry condition appraised the infant's temperament as more negative and appraised the infant as having less positive intent than those in the babble condition. Sound condition did not significantly predict appraisal of infant negative intent. No interactions between trait anger and sound condition on appraisals of the infant were found; therefore, only main effects have been reported.

**TABLE 4:** Linear regressions assessing trait anger and sound condition as predictors of appraisals of the infant (*n* = 244).

Variables	Model 1				Model 2			
	*β*	95% CI		*p*	*β*	95% CI		*p*
		LL	UL			LL	UL	
Negative temperament								
Trait anger					**0.12**	**0.03**	**0.20**	**0.008**
Cry (vs. babble)	**1.47**	**1.28**	**1.66**	**< 0.001**	**1.46**	**1.27**	**1.66**	**< 0.001**
Negative intent								
Trait anger					0.09	−0.05	0.22	0.198
Cry (vs. babble)	0.25	−0.01	0.52	0.060	0.25	−0.02	0.51	0.066
Positive intent								
Trait anger					−0.02	−0.11	0.07	0.702
Cry (vs. babble)	**−1.43**	**−1.60**	**−1.27**	**< 0.001**	**−1.43**	**−1.60**	**−1.27**	**< 0.001**

*Note:* Model 1 included sound condition as a predictor. Model 2 included trait anger and sound condition as predictors. Both models adjusted for characteristics that differed by sound condition (i.e., number of children) and the cognitive test the participant completed. Participants allocated to the pink noise condition were not exposed to the sound of an infant and thus were not asked to appraise any infant. Bolded rows indicate significance < 0.05.

Abbreviations: CI, confidence interval; LL, lower limit; UL, upper limit.

#### 3.2.3 Cognitive Performance

In linear regressions, there were no effects of trait anger and sound condition on cognitive performance in the Navon task, go/no‐go task, or the RMET‐R (see Table 5). There were also no interaction effects between trait anger and sound condition on any indicator of cognitive performance; therefore, only main effects have been reported.

**TABLE 5:** Linear regressions assessing trait anger and sound condition as predictors of task performance (*N* = 368).

Variables	Model 1				Model 2			
	*β*	95% CIs		*p*	*β*	95% CIs		*p*
		LL	UL			LL	UL	
Navon task latency difference (ms), *n* = 114								
Trait anger					0.02	−0.15	0.20	0.787
Cry (vs. babble)	−0.41	−0.85	0.04	0.075	−0.41	−0.85	0.04	0.076
Cry (vs. pink noise)	−0.08	−0.52	0.36	0.720	−0.08	−0.52	0.36	0.722
Go/no‐go commission errors (%), *n* = 118								
Trait anger					0.12	−0.05	0.30	0.170
Cry (vs. babble)	−0.19	−0.58	0.20	0.345	−0.17	−0.54	0.21	0.375
Cry (vs. pink noise)	0.17	−0.16	0.51	0.306	0.18	−0.17	0.53	0.319
RMET‐R total correct responses, *n* = 136								
Trait anger					0.09	−0.08	0.27	0.294
Cry (vs. babble)	0.13	−0.29	0.56	0.533	0.14	−0.28	0.56	0.514
Cry (vs. pink noise)	−0.27	−0.66	0.13	0.181	−0.24	−0.63	0.15	0.226

*Note:* Model 1 included sound condition as a predictor. Model 2 included trait anger and sound condition as predictors. Both models adjusted for characteristics that differed by sound condition (i.e., number of children).

Abbreviations: CI, confidence interval; LL, lower limit; ms, milliseconds; RMET‐R, Reading the Mind in the Eyes Test—Revised; UL, upper limit.

## 4 Discussion

Using an experimental study design, we investigated the role of father trait anger and exposure to infant cry on factors crucial to fathers' capacity to provide sensitive caregiving. Hypotheses were partially supported. Specifically, we found that both trait anger and infant cry were uniquely associated with increased likelihood of fathers' experiencing angry emotional states and making negative appraisals of infants, but contrary to hypotheses, we found no effects on cognitive performance. Hypotheses that exposure to infant cry would compound effects of trait anger on the measured outcomes were not supported. Trait anger and exposure to distressed infants are both risk factors for father angry emotional states in the parenting environment and negative appraisals of infants and may be relevant to identifying and targeting interventions for expectant and new fathers.

### 4.1 Angry Emotional State

In our study, as fathers' levels of trait anger increased, so too did their post‐exposure angry emotional state, including feelings of anger, feeling like yelling at someone, and feeling like hitting someone. This effect was evident across all sound conditions, indicative of a cross‐situational impact of disposition on emotional states. This finding contributes to the extensive body of research where measurement of a dispositional tendency to feel angry is effective in predicting later in the moment feelings of anger (Veenstra et al. 2018).

Overall, fathers exposed to infant crying, compared to controls, were also much more likely to feel angry and feel like yelling at someone; however, infant crying was not significantly associated with higher rates of feeling like hitting someone. The finding that exposure to the cry condition would predict higher post‐exposure feelings of anger than the control conditions was in line with prior research in mothers where, in an experimental investigation, extended exposure to the sound of a crying infant was associated with more post‐exposure feelings of frustration and negative emotions (Fairbrother et al. 2015) and in research on parents where qualitatively parents reported feeling anger in response to their own crying infant (for a review, see: Muller et al. 2023). Further, the finding that exposure to infant cry was not significantly associated with feeling like hitting someone may at first appear encouraging. However, the effect sizes for associations with trait anger and infant crying compared to controls were similar. This warrants further investigation with regard to which fathers would be most susceptible to physical expressions of anger in the context of hearing an infant cry.

While both trait anger and infant cry independently predicted angry emotional states, the sound of an infant crying did not exacerbate the effects of trait anger. We theorized that in the presence of a crying infant that fathers with higher levels of trait anger may be less able to provide sensitive care and therefore be less likely to soothe the infant, resulting in continued and potentially amplified feelings of anger. One explanation for the lack of interaction effect between trait anger and sound condition on angry emotional state may be that all fathers in this study were not given the opportunity to attempt to soothe the crying infant and therefore were exposed to the feedback loop of anger rumination that in the parenting environment may be more likely experienced by fathers with elevated trait anger. Future research should explore the potential for an interaction effect in a naturalistic setting. However, the associations between trait anger and infant cry with angry emotional state suggest that consideration should be given to intervening on the regulation of trait‐based feelings and the expression of emotion, and also indicate there are likely benefits from supporting expectant fathers with strategies to regulate emotion in the context of infant distress for situations where they are unable to resolve their infant's cry. Interventions to modify traits have had some success (Roberts et al. 2017); however, it is not currently clear why interventions to reduce feelings of anger are not effective for all participants (Lee and DiGiuseppe 2018; Shepherd and Cant 2020). Regarding support for fathers, there are few evidence‐based interventions that involve emotion regulation (e.g., Rayburn and Coatsworth 2021). However, some evidence is emerging for father‐inclusive programs that target responses in the context of infant distress, such as the ICON program, for which the efficacy is currently under investigation (Brose 2023).

### 4.2 Appraisals of Infants

Fathers with higher levels of trait anger appraised the recorded infant as more negative in temperament than fathers with lower levels of trait anger, irrespective of whether they were exposed to the infant cry or babble conditions. Prior research investigating appraisals of crying infants in nonparents found that post‐exposure feelings of anger were associated with more negative appraisals of infant temperament (Cohen‐Bendahan et al. 2014). In investigating pre‐exposure trait anger, we extended upon this prior research, finding that dispositional anger is related to negative appraisals of infant temperament. Given the stability of trait anger (Rebollo and Boomsma 2006), this suggests a potential prospective indicator for risk and target for prevention of father negative perceptions about infants. Additionally, as expected, those who were exposed to the infant crying condition appraised the infant as more negative in temperament than those exposed to the infant babble condition. However, exposure to the infant cry condition did not exacerbate effects of trait anger on negative appraisals of the infant's temperament.

Trait anger was not associated with fathers appraising infants as having negative intent or less positive intent. This absence of an impact of father trait anger was contrary to expectations, given prior links between trait anger and a tendency to attribute hostile intent to others (Li and Xia 2020; Wilkowski and Robinson 2008). Further, in previous qualitative literature, fathers with elevated levels of anger have made hostile attributions about infant intent, for example, “*… is this baby trying to play me up or something, on purpose*?” (Parfitt and Ayers 2012). This finding also contrasts with existing literature, that in childless young adults, anger was associated with increased appraisals of a crying child as being more intentionally difficult (Cohen‐Bendahan et al. 2014). One explanation may be that once in the fathering role and after gaining experience with infant behavior, fathers with higher levels of trait anger develop an ability to make appraisals of infant intent that are less susceptible to in the moment infant behavior. These findings suggest that other factors may be more relevant to these attributions, for example, levels of reflective functioning capacity (Rutherford et al. 2017). Results in the current study encouragingly suggest that father trait anger may not permeate appraisals of infant intent, which is a component of healthy and strong father‐infant bonds (Condon et al. 2008). Further, fathers exposed to infant cry did not appraise the infant as having more negative intent than a babbling infant, but did appraise the crying infant as having less positive intent. These findings may reflect a nuanced appraisal of infant behavior by fathers, such that a cry is appraised as a natural form of communication to express the infant's needs, whereas a babble is appraised as an attempt to engage positively with a caregiver (LaGasse et al. 2005).

### 4.3 Cognitive Performance

We did not detect an effect of trait anger on markers of cognitive performance. While the finding that trait anger was not associated with cognitive performance may be an indication of a true null effect, it may also be the case that an association between trait anger and cognitive performance is only apparent for those with high levels of trait anger. For example, in previous literature, a relationship between trait anger scores and impulse control was found in a sample with clinically high levels of trait anger (Lievaart et al. 2018), but not in a community sample (Lievaart, van der Veen, et al. 2016). Further, despite inducing anger in those exposed to the infant cry condition, we did not detect any effect of sound condition on markers of cognitive performance. This finding aligns with a new study by Waizman et al. (2024) which investigated the impact of infant cry on fathers' impulse control. One explanation for this lack of a difference between sound conditions on cognitive performance may be due to the presence of a sound stimuli in all conditions; it may be that listening to any sounds might serve as an auditory distraction that impacts cognitive performance. However, Waizman et al. (2024) found no difference on fathers' impulse control performance between a silent condition and two sound conditions (infant cry and pink noise). Therefore, it remains possible that this non‐significant relationship between exposure to infant cry and cognitive performance reflects a true effect, and that fathers, who have had the life experience of being exposed to the sounds of a distressed infant, have built tolerance to infant cry, or have identified strategies to minimize or mitigate the effects of infant cry on their cognitive abilities. This is supported by previous literature where, while the sound of an adult woman crying negatively impacted men's cognitive performance, the sound of an infant crying did not (Rigo et al. 2017). However, it is also possible that the tasks were not sensitive enough to exhibit differences in performance particularly remote, online testing of cognitive performance. For example, the RMET‐R involves reading and therefore necessitates that participants can take as long as they need to complete each item. Thus it may be that effects are less apparent when participants are given unlimited processing time, even in the presence of a crying infant. Future research may detect cognitive performance effects with tasks that place greater demands on participants' cognitive resources.

### 4.4 Limitations and Future Directions

A limitation of this study is that subsample sizes within combinations of cognitive task assignment and sound condition assignment may have been underpowered to detect clinically relevant (i.e., small) interaction effects between trait anger and sound condition on the cognitive performance outcomes. This is the first research in the field to investigate these potential interaction effects, and future research should seek to replicate these effects.

While we were able to control some facets of the participant experience by including checks that participants were using headphones and testing to ensure that there were no differences between condition groups on the environment that participants were in when participating in the study (i.e., whether participation was conducted alone), we were not able to account for other potential environmental confounders (e.g., competing household or workplace demands). These potential confounders may have impacted participants' performance of the cognitive tasks and may have obscured potential effects of trait anger or sound condition on cognitive performance. While we did not find any differences between groups on self‐reports of these environmental conditions, future research should nevertheless seek to replicate these findings in the laboratory environment where potential environmental confounders can be controlled. A laboratory environment would allow for comparison between infant cry and a silent control group, helping to disentangle the effects of cognitive testing from exposure to infant‐related sounds. Further, laboratory assessment would also have allowed for assessment of physiological indicators of anger.

The generalizability of our findings may also be impacted, as fathers' reactions to their own infants' real‐time cries, communicating to them in the moment of need, may differ from how they respond to the recording of a non‐related infant's distress. To mitigate this potential impact, we used validated sound recordings which had been curated by Barr et al. (2014) to ensure only genuine cry or babble sounds were retained. In their sample of mothers, the 10‐min infant cry recordings resulted in an initial increase in frustration in the first 100 s followed by a gradual increase in frustration throughout the experiment and a post‐exposure increase in anger (Barr et al. 2014). However, we acknowledge that our preexperimental information to participants revealed the duration which participants would be exposed to the sounds, which was a point of methodological difference from Barr et al. (2014). In natural settings, the unpredictability of the duration of an infant's cry may impact fathers' responses, and therefore the disclosure of the duration of the sound to participants may have impacted the generalizability of the findings.

Moreover, this sample was not limited to only fathers of infants and included fathers with a youngest child up to 10 years of age, with only 12% of the sample reporting having an infant 1 year of age or younger. Postpartum fathers may be more reactive to infant crying than fathers of older children or non‐fathers. For example, in previous research, postpartum fathers reported higher levels of frustration than prepartum fathers when listening to prolonged infant cry (Drabkin et al. 2024). We did not have sufficient power to analyze the effects of trait anger on fathers' responses to infant cry across different stages of their parenting experience. Future research should explore these potential variations.

### 4.5 Implications

Our research presents three key insights. The first is that both fathers with elevated levels of trait anger and fathers exposed to infant distress are likely to have more negative responses to infants. The second insight is that it is erroneous to assume that these effects compound, thereby leading fathers with elevated trait anger to respond more adversely to infant crying than fathers in general. Therefore, while most fathers may benefit from support to modulate their emotional responses to infant distress and negative appraisals of distressed infant temperament, fathers with higher levels of trait anger may require intervention with a specific focus on managing their emotional responses generally, even in the context of positive infant interactions. It is critical to implement supports and interventions early, as prior research demonstrates that father trait anger affects bonding with infants and parenting experiences in the toddler years (Francis et al. 2023, 2024) and that when distressed infants are not able to be successfully settled, sleep quality is compromised, which has cascading effects on relationship outcomes, mental health, and work safety (Macdonald, Graeme, et al. 2021; Wynter et al. 2019, 2020). It is important to target these supports directly to fathers, as maternal‐focused services pose barriers to fathers' engagement in the perinatal healthcare system (Macdonald et al. 2022). The third insight is the promising finding that the cognitive processes linked to fathers' ability to attend to their infants' needs may not necessarily be impacted by either trait anger or exposure to infant cry. This insight is supported by recent literature which found no effect of infant cry on father cognition (i.e., impulse control; Waizman et al. 2024). However, the samples of both the current study and Waizman et al. (2024) were community‐based, and therefore more research is needed to establish this finding in clinical populations which may potentially be more vulnerable to impacts on their cognitive performance.

## 5 Conclusions

This study experimentally investigated the effects of father trait anger and exposure to infant cry on fathers' angry emotional state, appraisals of infants, and cognitive performance. We found that exposure to infant cry did not exacerbate the impact of trait anger on any outcome. Trait anger predicted angry emotional state post‐exposure and more negative appraisals of infant temperament. Fathers exposed to the sound of a crying infant were more likely to feel angry post‐exposure than fathers exposed to infant babble or non‐infant‐related control noise. Further, the crying infant was appraised more negatively than the babbling infant. Interventions which aim to develop expectant fathers' abilities to regulate their emotional responses may aid in reducing angry emotional states and negative appraisals of infant temperament, particularly for fathers with elevated levels of trait anger. Additionally, there are likely also benefits to fathers' capacity to provide sensitive responses in supporting fathers with strategies to regulate emotion in the context of infant distress. We did not find any impact of trait anger or infant cry on cognitions important to the provision of sensitive responses, which is a promising but preliminary finding.

## Author Contributions

Study conception and design: Lauren M. Francis, Jacqui A. Macdonald, Peter G. Enticott, and Ashlee Curtis. Preparation of data collection materials (i.e., survey and experiment development): Lauren M. Francis and Bridgette E. Speranza. Analysis and interpretation of results: Lauren M. Francis, Liam G. Graeme, Jacqui A. Macdonald, Peter G. Enticott, and Ashlee Curtis. All authors reviewed and approved the manuscript.

## Ethics Statement

Ethics approval was granted by Deakin University Human Research Ethics Committee (DUHREC), project identifier: DUHREC 2021‐134.

## Conflicts of Interest

The authors declare no conflicts of interest.

## Supporting information


File S1.

File S2.

File S3.

Table S1.

Table S2.

## Data Availability

Data and code are available at https://doi.org/10.17605/osf.io/x24az.
