# Supplementary material for: Father Trait Anger and Exposure to Infant Cry: Effects on Emotion, Appraisals of Infants, and Cognitive Performance
Source: J Pers. 2025 May 23;94(2):264–76. doi: 10.1111/jopy.13029 (PMC12988345; doi:10.1111/jopy.13029)
Supplement: Supplementary file 1 — File S1. [file JOPY-94-264-s001.docx]

Supplementary 1

Hypothesis and Analyses Adapted from Pre-Registration

One hypothesis (and corresponding analyses) was adapted from pre-registration. The pre-registered hypothesis only included participants within the infant cry condition, and is quoted here:

“*We hypothesise that anger will be induced for participants exposed to the infant crying condition, such that participants in the infant crying condition will demonstrate higher average scores on state anger related indicators of emotional state post-exposure compared to pre-exposure.”*

We adapted this hypothesis to include participants in all sound conditions and to align with the other hypotheses in the pre-registration (also included in the manuscript) which additionally included trait anger as a predictor. The adapted hypothesis as it appears in the manuscript is quoted here:

“*We first hypothesized that trait anger and exposure to infant cry, relative to control conditions, would each be positively associated with post-exposure feelings of anger, feeling like yelling at someone, and feeling like hitting someone and that effects of trait anger would be stronger for fathers in the infant cry condition.*”

The pre-registered analyses to test this hypothesis is quoted here:

“Experimental manipulation checks will be conducted to test for inducement of anger in the infant crying condition. Specifically, pre-test scores on any of the emotional states that indicated distress or anger (i.e., ‘distressed’, ‘irritated’, ‘like yelling at somebody’, ‘like hitting something’, ‘angry’, or ‘agitated’) will be compared with dependent samples t-tests to post-test scores on the same measure for participants in the infant crying condition.”

We adapted the analyses used to this test this hypothesis in alignment with the analyses used to test the other pre-registered hypotheses. The adapted analysis as it appears in the manuscript is quoted here:

*“A series of regression analyses were conducted to investigate main and interaction effects of trait anger and sound condition on key outcomes. Outcome variables were post-exposure angry emotional state (feeling angry, feeling like yelling at someone, and feeling like hitting someone)…* *Specifically, robust regressions (linear or logistic as appropriate) were estimated in three sets in which each outcome variable was regressed first onto sound condition, second onto trait anger and sound condition (entered together), then third onto trait anger, sound condition, and the interaction between those two predictors.”*
