# Supplementary material for: Father Trait Anger and Exposure to Infant Cry: Effects on Emotion, Appraisals of Infants, and Cognitive Performance
Source: J Pers. 2025 May 23;94(2):264–76. doi: 10.1111/jopy.13029 (PMC12988345; doi:10.1111/jopy.13029)
Supplement: Supplementary file 2 — File S2. [file JOPY-94-264-s004.docx]

**Supplementary 2**

**Data Quality Checks**

***Headphone Check Procedure***

Participants were required to use headphones as this allows for regulation of the experience of the audio stimuli and reduces differences in participants experiences including background noise (Woods et al., 2016). To ensure that participants were using headphones and had volume set to an appropriate level to hear the audio conditions they were required to complete the Headphone Check procedure (Woods et al., 2016; Millisecond Software, 2022). The task required participants to adjust the audio volume loud enough that they could hear three tones played aloud. Participants were then asked to identify the loudest tone, repeated for a total of six tests. Due to phase-cancellation (i.e., the waves of two sounds cancelling each other out) this task is very difficult to complete over loudspeakers, but easy to complete with headphones (Woods et al., 2016). Participants who failed the Headphone Check Procedure (completing less than five of six the tests correctly; Woods et al., 2016) were not able to continue participation (*n* = 157) and were streamed out of the survey to a message that explained that they had failed the Headphone Check Procedure and could no longer participate. This condition of participation was outlined to all participants in the Plain Language Statement.

***Attention Check***

To ensure that participants were providing thoughtful responses to self-report items two attention checks were implemented to detect careless responding in the questionnaire component of the experiment. The attention checks were adapted from Müller et al. (2022). After item five of the trait anger measure, the following item was added: “If you are paying attention, select ‘Sometimes’, otherwise you will be disqualified”. This item offered the same response options as the trait anger measure. The Reflective Functioning Questionnaire (RFQ; not included in the current paper) was also asked pre-cognitive testing. Within the RFQ item matrix, after item two, the following item was added: “If you are paying attention, select ‘Strongly agree’, otherwise, you will be disqualified”. This item offered the same response options as the RFQ. Participants who did not pass either of the attention checks (*n* = 7) were not included in the analytic sample (as per Müller et al., 2022) and were streamed out of the survey to a message that explained that they had failed an attention check and could no longer participate. This condition of participation was outlined to all participants in the Plain Language Statement.

***Audio Check***

To ensure that participants were keeping headphones on for the duration of the cognitive testing, an audio check was implemented. Immediately after completing the cognitive test, participants were displayed a “Please wait” message for 5 seconds. While this was presented an audio played asking them to “Remember the colour Purple”. This audio was generated with English (US) Siri voice 4, Played aloud on iPhone XR, and recorded on a Google Pixel 6. After the 5 seconds elapsed, participants were-redirected to a questionnaire, where the first item was “Please select the colour that you were asked to remember during the task” with response options: Red, Orange, Yellow, Green, Blue, Purple, Pink, and ‘I didn’t hear a colour stated’. Data from participants who did not select purple (*n* = 14) were excluded from analyses to preserve the integrity of the data.

***Cognitive Testing Data Quality Checks***

All the cognitive testing participant exclusions were conducted as per the pre-registration. Participants who completed the go/no-go task were excluded if they had a frequency of omission errors more than three standard deviations above the mean (i.e., participants waiting too long to respond). Participants who completed the Navon task were excluded if they had either a frequency of omission errors more than three standard deviations above the mean or a response time three standard deviations above or below the mean. Participants who completed the RMET-R were excluded if they selected the word in the same spatial region for every trial within a block (i.e., not moving the mouse and skipping through the trials by selecting the same option for each trial) or if their response time was more than three standard deviations below the mean (i.e., responding before they had time to consider the response options).

An additional (not pre-registered) exclusion criteria was applied. Participants who completed the Navon task were also excluded if 0% of their responses in a condition were correct. The Navon task involves two conditions, with instructions presented at the beginning of each condition instructing participants to swap from a global focus to a local focus (or local to global, depending on which was completed first). Completing a condition with 0% correct responses indicates that participant did not read the instructions correctly and were performing the task incorrectly.

**References**

Millisecond Software (2022). Inquisit 6 headphone check procedure. Available at: https://www.millisecond.com/download/library/headphonecheck.

Müller, S., Wendt, L. P., Spitzer, C., Masuhr, O., Back, S. N., and Zimmermann, J. (2022). A critical evaluation of the reflective functioning questionnaire (RFQ). *J. Pers. Assess.* 104, 613–627. doi: 10.1080/00223891.2021.1981346.

Woods, K. J. P., Siegel, M., Traer, J., and McDermott1, J. H. (2016). Headphone screening to facilitate web-based auditory experiments. *Atten Percept Psychophys.* 176, 100–106. doi: 10.3758/s13414-017-1361-2.
