# Supplementary material for: Father Trait Anger and Exposure to Infant Cry: Effects on Emotion, Appraisals of Infants, and Cognitive Performance
Source: J Pers. 2025 May 23;94(2):264–76. doi: 10.1111/jopy.13029 (PMC12988345; doi:10.1111/jopy.13029)
Supplement: Supplementary file 3 — File S3. [file JOPY-94-264-s003.pdf]

Supplementary 3.

Flow Chart Summary of Participant Exclusions.

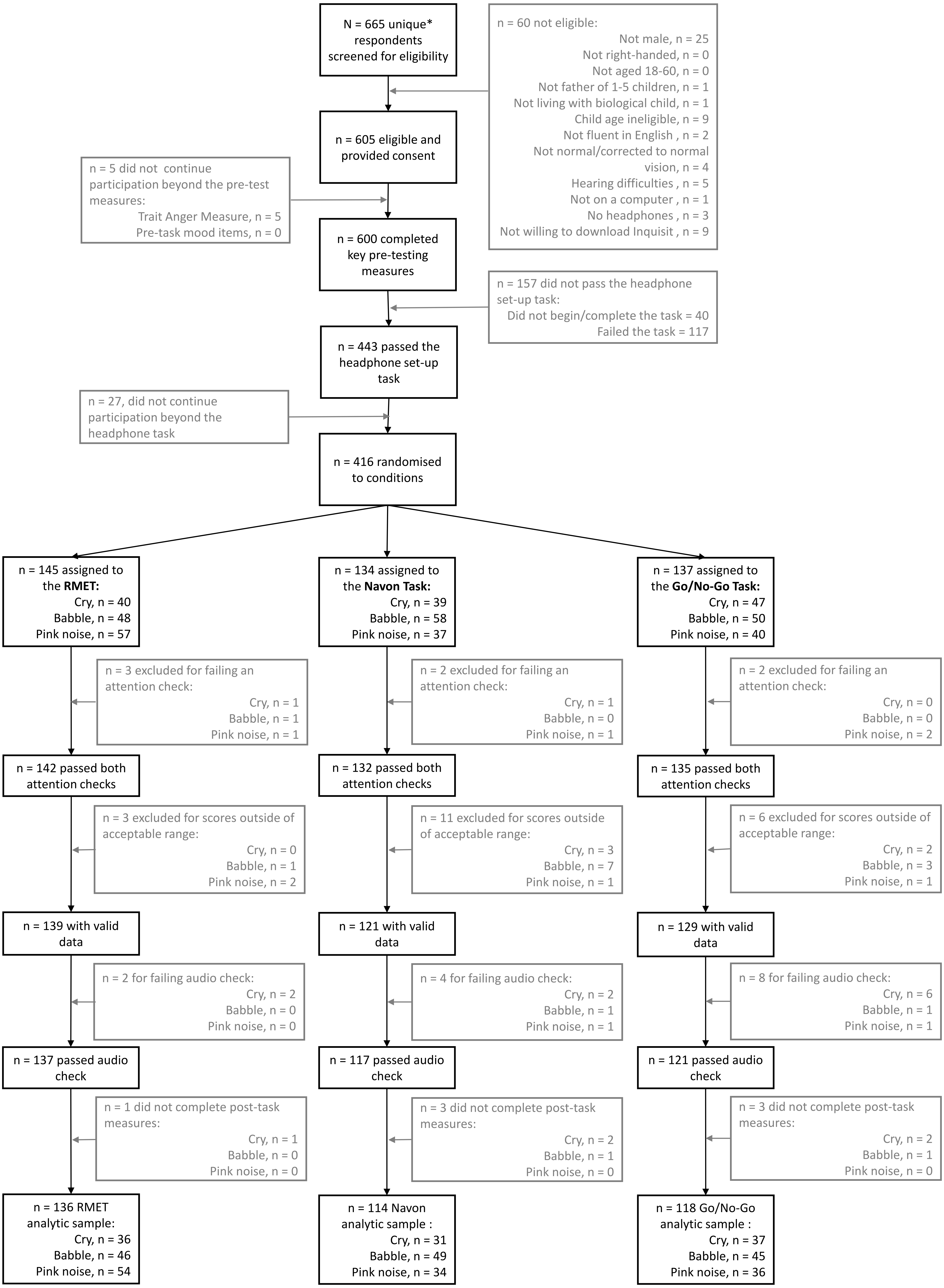

Note. Attention checks were included in survey before randomisation occurred, however participants were excluded after completing the full study and therefore have been included here as excluded after randomization; RMET = Reading the Mind in the Eyes Task; *Duplicates participants were removed before this step.
